# One‐Pot Synthesis and Characterization of Magnetic α‐Fe_2_O_3_/CuO/CuFe_2_O_4_ Nanocomposite for Multifunctional Therapeutic Applications

**DOI:** 10.1002/open.202400277

**Published:** 2024-10-30

**Authors:** Fahmida Akter Sahara, Mst. Sabiha Sultana, Md. Khairul Amin, Muhammad Shamim Al Mamun, Palash Kumar Dhar, Sagar Kumar Dutta

**Affiliations:** ^1^ Chemistry Discipline Khulna University Khulna 9208 Bangladesh; ^2^ Agrotechnology Discipline Khulna University Khulna 9208 Bangladesh

**Keywords:** Nanocomposite, Antifungal agents, Antioxidants, Drug delivery, Release kinetics

## Abstract

This study demonstrates a novel nanostructured drug delivery system utilizing α‐Fe_2_O_3_/CuO/CuFe_2_O_4_ ternary nanocomposite for effective drug transport in sick tissues. *Centella Asiatica* plant extract was employed to synthesize the Fe_2_O_3_/CuO/CuFe_2_O_4_ nanocomposite via sol‐gel auto combustion technique. The structural and morphological characteristics of the nanocomposite were investigated by XRD, FT‐IR, SEM, EDX, and VSM for magnetic properties. The XRD analysis demonstrates the successful synthesis of Fe_2_O_3_/CuO/CuFe_2_O_4_ nanocomposite with an average crystallite size of 18.393 nm. The antioxidant and antifungal capabilities of this nanocomposite were assessed for its biological activity. A notable inhibitory zone was observed when tested against *the Alternaria spp*. and *Bipolaris sorokiniana* fungi. An IC_50_ value of 109.88 μg/ml was found in the DPPH test, indicating that the nanocomposite exhibited remarkable antioxidant characteristics. Subsequently, metronidazole was encapsulated with a success rate of 55.53 % at pH 1.2, while at pH 7.4 it gained 57.83 %. The drug release of nanocomposite at pH 1.2 after 330 min was 43.41 % and at pH 7.4 after 300 min it was 52.3 %. The results indicate its potential as an excellent candidate for drug delivery. Furthermore, pH was found to be an effective catalyst in the drug loading and release processes.

## Graphical abstract

This article presents the sol‐gel auto combustion synthesis of α‐Fe_2_O_3_/CuO/CuFe_2_O_4_ using *Centella Asiatica* plant extract as a green precursor. Significant antifungal and antioxidant properties were shown by this compound. This nanocomposite also investigated as a drug nanocarrier at different pH levels and compared by fitting the data to various drug release kinetics models.

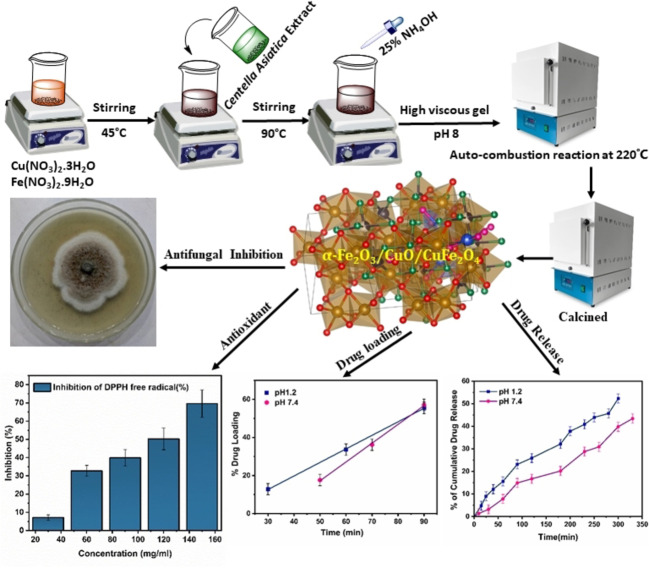

## 1 Introduction

In today's world, the advancement of novel drug delivery systems (DDS) is providing a more effective means of introducing medicinal compounds into the body in a more controlled manner to improve their efficacy and safety. The emergence of nanotechnology has brought about revolutionary shifts in medicine and DDS. The simplistic physicochemical characteristics and small size of nanoparticles help to achieve lower dosages, larger biocompatibility, cost‐effectiveness, scalability, reduced toxicity, and localization of therapeutic molecules at disease sites.[^1^, ^2^, ^3^]

The magnetic properties of nanoparticles have enabled a new generation of drug delivery technology due to the responsiveness of their metallic formulations. Targeting drug‐loaded nanoparticles to active sites utilizing an external magnetic field is straightforward.[^4^, ^5^] Inorganic nanoparticles exhibit stronger magnetic properties compared to other types, such as polymeric or lipid nanoparticles.[^6^, ^7^, ^8^] Along with magnetic properties, their high chemical stability, non‐immunogenicity, ease of surface functionalization, and tunable structure support various biomedical applications such as bioimaging, targeted gene delivery, hyperthermia for cancer treatment, and biomedical sensing..[^9^, ^10^, ^11^] Different types of inorganic nanoparticles have been exploited recently, including gold nanoparticles, metallic nanoparticles, mesoporous silica nanoparticles, and carbon nanoparticles (fullerenes, carbon nanotubes, and graphene).[^9^, ^12^] Radulescu et al. 2023 and Salih et al. 2023 noted that magnetically active oxides and other iron‐based spinel oxide nanoparticles are frequently used because of their high electrical resistivity, mechanical hardness, plasmonic characteristics, and other properties.[^13^, ^14^] Specifically, α‐Fe_2_O_3_ ^[15]^ CuO,^[16]^ and CuFe_2_O_4_ ^[17]^ possess excellent structural, electrical, and magnetic properties, making them suitable for biomedical applications. Iron, copper, and oxygen are important physiochemical elements in our bodies, playing interconnected roles in maintaining essential body functions. Nanoparticles can be degraded and cleared from circulation by endogenous pathways.^[9]^ Due to their ease of implementation for nanocarrier applications, much study has been done in this field.

Along with the properties described, DDS should be biologically safe, have regulatory approval, and exhibit an enhanced therapeutic index. Singh et al. 2013 reported that Fe_2_O_3_ nanoparticles up to 2000 mg/kg body weight did not show genotoxicity in rats.^[18]^ Yan et al. 2023 stated the no‐observed‐adverse‐effect level (NOAEL) dose of Fe_2_O_3_ nanoparticles administered orally to rats for 94 days was 500 mg/kg. There were no notable differences in various health parameters, organ weights, and organ‐related measures between the treatment groups and the control group.^[19]^ Gopinath et al. 2016 described that cytotoxicity assay and apoptotic analyses proved the CuO nanoparticles are relatively cytofriendly to human mesenchymal stem cells and also demonstrate anti‐cancer activity on human adenocarcinoma AGS cell lines.^[20]^ Jasim et al. 2022 stated CuFe_2_O_4_ nanoparticles demonstrated low toxicity against rat pheochromocytoma (PC12) neuronal cells, with an IC_50_ value of 225.01 μg/mL based on cytotoxicity assay.^[21]^

Inorganic nanoparticles exhibit a promising source of antifungal activity to address the growing threat of fungal infections, drug resistance, and potentially have lower toxicity compared to traditional antifungal drugs.^[22]^ The antifungal mechanism inhibits mycelial growth and budding processes, degrades the fungus cell wall, and disrupts fungus proteins, prevents the development of reproductive structures.^[23]^ Nanoparticles containing antioxidant properties have shown promising results in addressing the challenges associated with conventional antioxidants and have enabled the development of a new class of innovative antioxidants. These antioxidants have unique physicochemical properties, high tolerance to harsh microenvironments, and neutralize free radicals, thereby limiting bodily damage and preventing neurodegenerative conditions.^[24]^ When developed, DDS should be immune to pathogen attacks and possess antioxidant properties upon entering the body. α‐Fe_2_O_3_,^[25]^ CuO,^[26]^ and CuFe_2_O_4_ ^[27]^ are reported to have excellent antifungal and antioxidant properties.

Current research trends indicate that composites of metal oxides are superior to single nanoparticles because of their enhanced chemical and physical robustness, tolerance, structural integrity, and synergistic effect.[^28^, ^29^] α‐Fe_2_O_3_,^[30]^ CuO,^[31]^ and CuFe_2_O_4_ ^[32]^ have been used in many studies to synthesize various composite materials, yielding better biomedical application outcomes than single nanoparticles.

The synthesis method of a nanocomposite is crucial because it influences the functionality, purity, and quality of particles and environmental safety. The literature reports various nanocomposite synthesis techniques, including hydrothermal,^[33]^ micro‐emulsion,^[34]^ combustion,^[35]^ co‐precipitation,^[36]^ and sol‐gel methods.[^37^, ^38^] The sol‐gel auto‐combustion method is particularly suitable for solid‐state reactions as it allows precise controls over stoichiometry, phase formation, and particle size homogeneity. Its ease of use, cost‐effectiveness, lack of requirement for special equipment, and ability to be conducted at moderate temperatures are some benefits that led us to choose the sol‐gel process.[^39^, ^40^]

Green sources are used in synthesizing nanocomposites as reducing agents to minimize toxicity, enabling their successful application in biomedical‐related areas, such as drug delivery, without further alterations. These nanocomposites offer several advantages, such as minimal toxicity and biocompatibility, making them suitable for biological and pharmacological applications.^[41]^ Recent studies have explored the use of *Centella Asiatica* in the green synthesis of nanoparticles.[^42^, ^43^] It is a medicinal herb that has potential antioxidant, antimicrobial, restorative, detoxifying, and stimulant activities. It is used in the treatment of various skin conditions and has potential benefits for anxiety and cognitive function, effective in the treatment of wounds, hypertrophic scars, and burns, treating conditions such as cholera, jaundice, diarrhea, and hepatitis, promotes collagen synthesis, fibroblast proliferation, and improves skin tensile strength.[^44^, ^45^]

As reported earlier, the conventional method for synthesizing ternary nanocomposite is quite complicated and time‐consuming.[^46^, ^47^]

Therefore, this research aims to synthesize α‐Fe_2_O_3_/CuO/CuFe_2_O_4_ ternary nanocomposite using a one step sol‐gel auto combustion method with *Centella Asiatica* extract as a green source. The structural, morphological, optical, and magnetic characterizations, as well as its utility as a drug nanocarrier, have been studied. Its effectiveness as both an antifungal and antioxidant has been conclusively demonstrated, showing its capability to restrict the vital functions of pathogenic microorganisms. The drug loading and release profiles at different pH levels were examined and compared by fitting the data to various drug release kinetics models.

## 2 Materials ans Methods

### 2.1 Materials

All the chemicals used in this work were of analytical grade and used as received. Cu(NO_3_)_2_.3H_2_O, Fe(NO_3_)_2_.9H_2_O, 25 % ammonia, ethanol, 2,2‐diphenyl‐1‐picrylhydrazyl (DPPH), methanol, sodium chloride, sodium hydroxide, hydrochloric acid, metronidazole drug, pH buffer 1.2 and 7.4 were purchased from Sigma Aldrich, Merck, and potato dextrose agar (PDA) media was purchased from Hi media. For the antifungal activity test, the fungi used were from the collection of the plant protection lab, Agrotechnology Discipline, Khulna University, Bangladesh.

### 2.2 Instruments

In this work, x‐ray diffraction was carried out using a Philips X'pert PRO x‐ray diffractometer, and Cu K radiation (1.5418) was used to determine the phase purity of the generated nanocomposite at the Material Science Division of the Atomic Energy Center in Dhaka. SHIMADZU IR Spirit Fourier transform infrared spectrometer was used to study the bond formation of the materials. A UV‐1900i UV‐visible spectrophotometer (Shimadzu Co., Japan) was used for the optical analysis of the sample. To examine surface morphology and to establish the elemental composition, field‐emission scanning electron microscopy (FE‐SEM) and energy dispersive x‐ray (EDX) spectroscopy were done by the Sigma 300 SEM from Zeiss. A Microsense vibrating sample magnetometer (VSM) model EV7 was used to analyze the magnetic properties of the materials at the Atomic Energy Center in Dhaka, Bangladesh. A multiple water quality meter of model MM‐43X from DKK‐TOA Corporation was used for pH_PZC_ analysis.

### 2.3 Preparation of *Centella Asiatica* Extract

As a standard procedure,,[^33^, ^48^] the *Centella Asiatica* leaves were rinsed with deionized water and then dried in the shadow at room temperature for 28 days. Then, the dry leaves were ground to powder completely. Ten grams of finely powdered leaves were combined with 400 ml of distilled water and allowed to agitate at about 60 °C for two hours. Before filtrating out on filter paper to eliminate any solid stuff from the extraction, the resulting supernatant was centrifuged at 5000 rpm for 8 minutes.

### 2.4 Green Synthesis of α‐Fe_2_O_3_/CuO/CuFe_2_O_4_ Nanocomposite

α‐Fe_2_O_3_/CuO/CuFe_2_O_4_ ternary nanocomposite was made using a simple one step sol‐gel auto combustion synthesis technique.^[49]^ Cu (NO_3_)_2_.3H_2_O and Fe (NO_3_)_2_.9H_2_O were dissolved in 100 ml of water and stirred for 30 minutes at 45 °C to create a solution. When the solution became dark and clear, 100 ml of leaf extract, which worked as a capping agent, was added dropwise at a 1 : 1 ratio. Furthermore, dropwise, 25 % ammonia was added to push the pH to 8. Afterward, it was heated to 90 °C on a hotplate while continuously stirred and gradually evaporated to create a very thick gel. The obtained gel was re‐heated in a muffle furnace preheated at 220 °C. After completely removing all the water molecules from the mixture, the gel produced a quick, flameless auto‐combustion reaction that produced the nanocomposite. Using a mortar and pestle, the resulting powder was then thoroughly ground. Afterward, the powder was sintered for 6 hours at 600 °C and cooled to room temperature.^[38]^ The reaction takes place during the process are shown below:


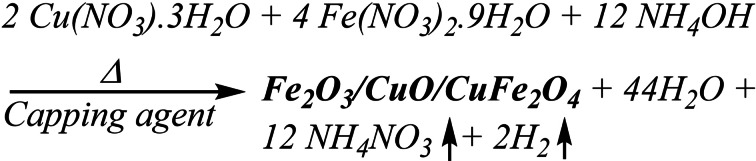


### 2.5 Antifungal Activity Assays

The effect of α‐Fe_2_O_3_/CuO/CuFe_2_O_4_ nanocomposite was tested against the mycelial growth of Alternaria spp. and Bipolaris sorokiniana. A sample ethanolic nanocomposite solution was used at a 2.033 mg/ml concentration.[^50^, ^51^] After adding the sample to Petri dishes containing PDA culture medium, a 5‐mm‐diameter PDA disc containing the corresponding strain's mycelium was placed in the center of the dish. The dish was then incubated at 28 °C for 30 minutes to allow ethanol to evaporate and the nanocomposite to diffuse. Every bioassay was carried out twice. Propiconazole fungicide was used as a control at 1 mg/ml concentration and under the same procedures as the nanocomposite. The mycelium growth was investigated after 9 days..^[52]^ The average diameter of the colonies was used to measure the impact of the nanocomposite.^[53]^ The percentage of mycelial growth inhibition is expressed using this formula.
(1)

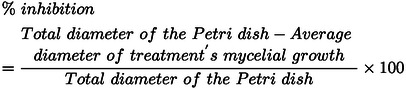




### 2.6 Free Radical Scavenging Activity

Free radical scavenging activity of Fe_2_O_3_/CuO/CuFe_2_O_4_ nanocomposite was calculated using the methodology of Saikia et al. (2010) with minor adjustments.^[54]^ The sample was mixed with 50 % methanol at various concentrations (30, 60, 90, 120, and 150 g/ml). A 0.15 % DPPH methanolic (50 %) solution was combined with those nanocomposite solutions of different concentrations in the ratio of 1 : 1 (2 ml from both). The mixture was then ultra‐sonicated by a bath sonicator and set aside for 15 minutes in the dark. A UV‐visible spectrophotometer was used to measure the reduction in absorbance of the resultant solution at 517 nm. DPPH was used as the control, and methanol worked as the blank. The percentage inhibition of synthesized nanocomposite's free radical scavenging activity was calculated using the following formula.^[55]^

(2) $$\%\;inhibition=\frac{\begin{array}{c} absorbance\;of\;the\;control-absorbance\;\\ of\;the\;sample \end{array}}{absorbance\;of\;the\;control}\times 100$$

### 2.7 pH_pzc_ Analysis

The following procedure was used to determine the pH at the point of zero charge (pH_PZC_) of Fe_2_O_3_/CuO/CuFe_2_O_4_ nanocomposite. Distilled water was used to make 0.01 M NaCl solution. Then, using 0.1 M NaOH or HCl solution as needed, six distinct standards with pH values ranging from 2 to 12 were adjusted. 0.05 g of the nanocomposite was added to 50 mL of each solution and agitated for 6 hours at room temperature. Furthermore, the final pH value of these liquids was taken. The pH_PZC_ was determined at the pH_initial_=pH_final_ value.^[56]^

### 2.8 Drug encapsulation

**Figure 1:**
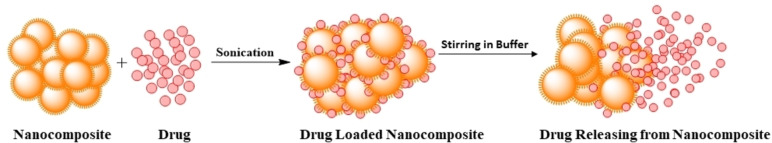
Schematic representation of drug loading and release mechanism.

Fe_2_O_3_/CuO/CuFe_2_O_4_ nanocomposite encapsulation efficiency (Figure 1) and the influence of pH on drug loading were examined at pH 1.2 and 7.4. The pH of the fasted‐state gastric fluid is 1.2 and the pH of the blood and intestinal fluid is 7.4.[^57^, ^58^] Here, Metronidazole was chosen as the model drug, because it is widely used in gastrointestinal treatments. However, its efficacy can be limited by rapid absorption.^[59]^ So, a nanocarrier would be an effective solution in this regard to maintain localization and controlled release. 20 mg metronidazole was mixed with an equivalent amount of nanocomposite in 100 ml of considered pH buffer. The mixture was sonicated for 1 hour at 37 °C. Using a UV‐vis spectrophotometer on absorbance peak at 320 nm, metronidazole concentration variation was noted in different time gaps until the change in concentration stopped. The quantity of added drug percentage encapsulated in the nanocomposite is known as encapsulation efficiency (EE).^[60]^

(3) $$\%\;Encapsulation\;efficiency=\;\frac{\begin{array}{c} Total\;drug\;\\ added-Free\;drug \end{array}}{Total\;drug\;added}\times 100$$

### 2.9 Drug Release

The drug release was calculated similarly to an earlier report by Bilas et al. 2017 with slight modifications.^[61]^ After loading the drug in the Fe_2_O_3_/CuO/CuFe_2_O_4_ nanocomposite, the solution was centrifuged for 15 minutes at 15000 rpm. Then, the separated particle was air dried for 2 days. After that, for the release study, the nanocomposite loaded with the drug was added to 100 ml of the required buffer and kept in constant stirring with a magnetic stirrer. In different time gaps, 2 ml of the buffer solution was withdrawn and replaced by 2 ml of fresh buffer (to maintain a constant volume). The concentration of the drug released at each time interval was observed using a UV‐vis spectrophotometer at an absorbance of 320 nm.^[62]^

(4) $$\%\;Cumulative\;Drug\;Release=\frac{Released\;Drug}{Total\;Drug\;Loaded}\times 100$$

### 2.10 Release Kinetics

To design a proper DDS, drug release kinetics (Table 1) optimization is required.

**Table 1:** Five different mathematical models to describe the drug release phenomena.

Equation No.	Kinetic Models	Regression Equation
5	Zero order kinetic model	Q=Qo+kt
6	First order kinetic model	logQ =logQ_0_+ $\frac{kt}{2.303}$
7	Higuchi model	Q=kt^1/2^
8	Korsmeyer–Peppas model	logQ=logk+nlogt
9	Hixson–Crowell model	Q_0_^1/3^ ‐ Q ^1/3^=kt

Here, Q is the total amount of drug released (mg/ml) at time t, Q_0_ is the initial amount of drug in solution (typically zero), t is the release time (min), and k is a constant.

In Equation (6), n is the release exponent indicating the drug release mechanism. If n =0.43, the drug release is referred to as Fickian diffusion (independent of drug concentration); if n =0.43 to 0.85, the drug release is referred to as non‐Fickian diffusion (anomalous transport), and if n >0.8, super case‐II transport and swelling for spherical particles are predicted.[^17^, ^63^, ^64^] Regression coefficients near unity indicate the best‐fit model for drug release kinetics.

## 3 Result and Discussion

### 3.1 Structural Study

**Figure 2:**
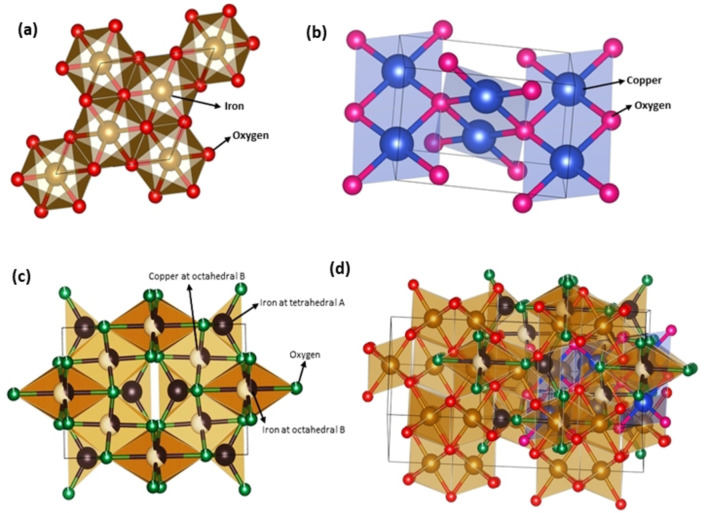
Structure of (a) α‐Fe_2_O_3_, (b) CuO, (c) CuFe_2_O_4_ & The proposed structure of (d) α‐Fe_2_O_3_/CuO/CuFe_2_O_4_ nanocomposite.

Fe_2_O_3_ stabilizes in the following phases: α (hematite), β (β‐Fe_2_O_3_), γ (maghemite), and meta‐stable ϵ (ϵ‐Fe_2_O_3_). The iron oxide which is most stable in the ambient atmosphere is α‐Fe_2_O_3_.^[65]^ Figure 2 **a** shows the rhombohedral structure of hematite, with space group R3c, which is of the corundum type, with symmetrical centers. Here, the oxygen lattice is closely packed, with two‐thirds of the octahedral sites within the framework of a hexagonal array occupied by Fe (III) ions.[^65^, ^66^, ^67^] Figure 2b depicts the copper atoms arranged in a planar square shape with four oxygen atoms in the CuO unit cell. Each Cu atom in CuO has four nearest neighbors among oxygen atoms and is located at the center of the oxygen rectangle. This proves that CuO has a monoclinic structure with space group C2/c.[^68^, ^69^]

CuFe_2_O_4_ can be crystallized in a cubic or tetragonal shape based on the preparation parameters. The catalytic activity of CuFe_2_O_4_ in its two crystalline forms differs, with the tetragonal form being more active than the cubic form.^[70]^ Jahn‐Teller distortion causes the cubic phase, which is stable at low temperatures, to shift into the tetragonal phase as the sintering temperature rises.^[71]^ This work attempts to make tetragonal CuFe_2_O_4_ as Figure 2c, where nearly all Cu^2+^ ions occupy the octahedral site in the inverse spinel structure of tetragonal CuFe_2_O_4_ (space group I4_1_/amd), while Fe^3+^ ions share both the tetrahedral A and octahedral B‐sites.[^72^, ^73^, ^74^] Figure 2 **(a, b, c)** is drawn with the help of COD ID #9015964, #1011148, and #9011012. The α‐Fe_2_O_3_/CuO/CuFe_2_O_4_ nanocomposite structure may be as in Figure 2d.

### 3.2 X‐ray Diffraction (XRD)

**Figure 3:**
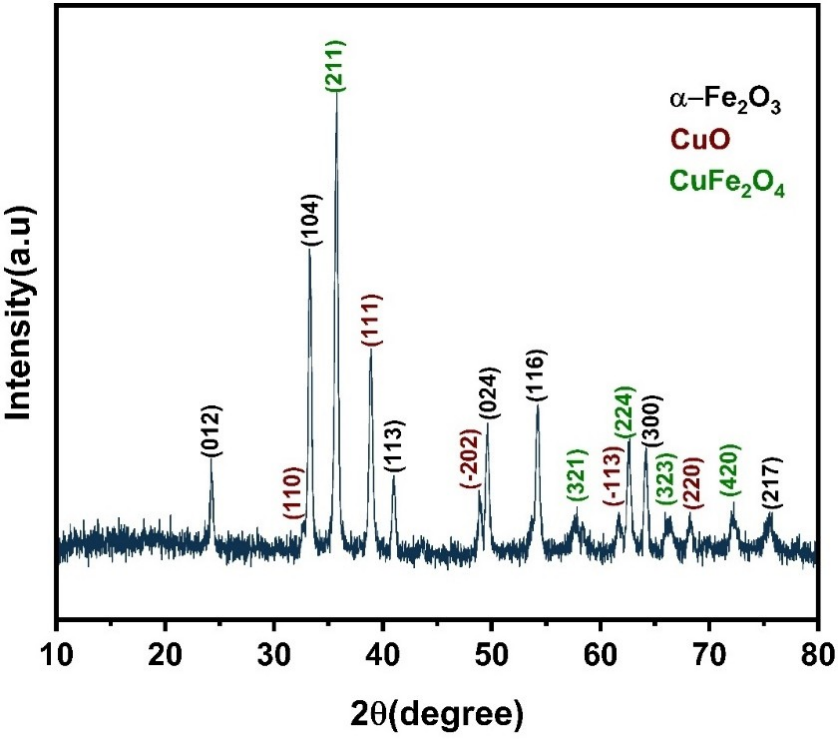
XRD pattern of α‐Fe_2_O_3_/CuO/CuFe_2_O_4_ nanocomposite.

The structural characterization of the α‐Fe_2_O_3_/CuO/CuFe_2_O_4_ nanocomposite was obtained through x‐ray diffraction, as depicted in Figure 3. The Fe_2_O_3_ patterns, which correspond to the (012), (104), (113), (024), (116), (300), and (217) planes, respectively, were found at 24.242, 33.27, 40.98, 49.57, 54.22, 64.11, 75.53 and they were consistent with the ICDD card (reference no: 01–084‐0307).^[75]^ So it has a rhombohedral structure with space group R3c. In accordance with the ICSD card (reference no: 00–041‐0254), the monoclinic CuO patterns were obtained at angles of 32.73, 38.84, 48.87, 61.59, 68.13, which correspond to the (110), (111), (−202), (−113) and (220) planes, respectively.^[76]^ The CuFe_2_O_4_ patterns, which correlated to the (211), (321), (224), (323), and (420) planes, were located at angles of 35.72, 57.73, 62.53, 66.14, and 72.08, respectively. This is consistent with the ICCD card (reference no: 34–0425) proves tetragonal CuFe_2_O_4_ synthesized, which has space group I4_1_/amd.^[77]^ The matching diffraction peaks with standard α‐Fe_2_O_3_, CuO, and CuFe_2_O_4_ show that the sol‐gel auto‐combustion approach was successful in obtaining ternary α‐Fe_2_O_3_/CuO/CuFe_2_O_4_ nanocomposites.

Nanocomposite size significantly influences determining material properties and performances due to their high surface area‐to‐volume ratio and quantum confinement effects.^[78]^ As the size changes to a high low, they exhibit altered physical properties, including lower melting points and unique optical adsorption, mechanical properties like superplasticity, enhanced toughness and strength, and improved chemical reactivity and stability.[^79^, ^80^] Their altering charge, surface shape, and solubility have a major impact on how they interact with cells and biomolecules.^[81]^ So they have a wide range of important applications including gas sensing, renewable energy, catalysis, electronics, medicine, diagnostics, drug delivery, cosmetics, construction, and food industry.^[11]^

The size of each domain in a unit cell's planes that coherently scatter light is known as the crystallite size. The Scherrer formula was used to determine the crystallite size by applying the full width at half maximum (FWHM) values of the corresponding peaks.

(5) $$D=\frac{k\lambda }{s\theta }$$

Here, λ is the x‐ray wavelength (0.1542 nm), β is the associated peak's full width at half maximum (in radians), θ is the Bragg's diffraction angle, and k is the Scherrer constant which is 0.89 for polycrystalline material. D is the average crystallite size (Table 2) which is calculated as 18.393 nm.

**Table 2:** Crystallite size calculation of α‐Fe_2_O_3_/CuO/CuFe_2_O_4_.

2θ	Miller indices (h k l)	FWHM in radian	Crystallite size (nm)	Average crystallite size (nm)
24.242	(012)	0.00436	30.7514	**18.393**
32.73	(110)	0.00663	19.8541	
33.27	(104)	0.00574	22.8999	
35.72	(211)	0.00574	22.7483	
38.84	(111)	0.00616	21.0079	
40.98	(113)	0.00600	21.411	
48.87	(−202)	0.00575	21.693	
49.57	(024)	0.00506	24.6166	
54.22	(116)	0.00593	20.5860	
57.73	(321)	0.03525	3.40902	
61.59	(−113)	0.00820	14.3712	
62.53	(224)	0.00541	21.6804	
64.11	(300)	0.00558	20.8260	
66.14	(323)	0.01762	6.52412	
68.13	(220)	0.00750	15.1485	
72.08	(420)	0.01204	9.21478	
75.53	(217)	0.00680	15.9386	

### 3.3 Fourier Transform Infrared Spectroscopy (FT‐IR)

**Figure 4:**
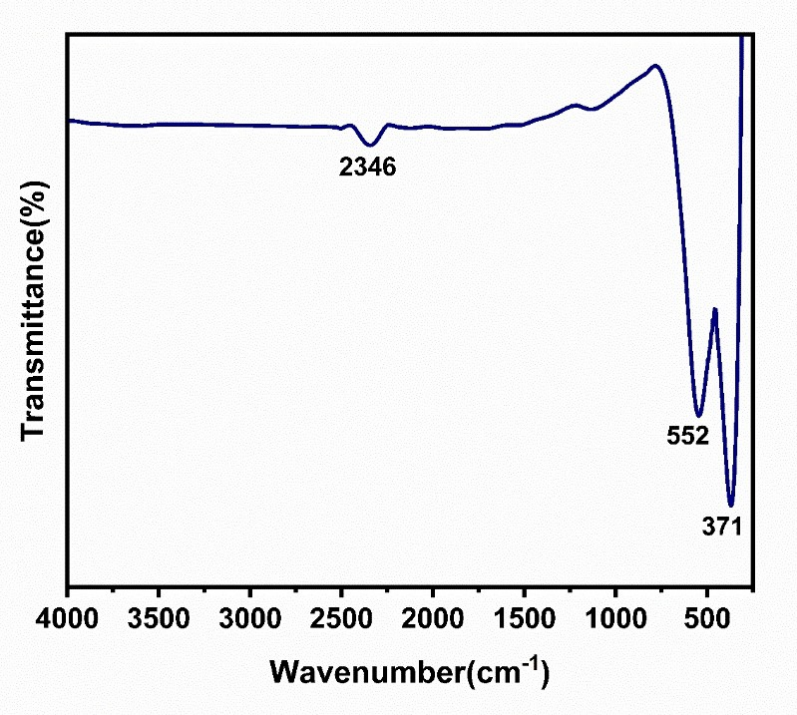
FT‐IR spectrum of α‐Fe_2_O_3_/CuO/CuFe_2_O_4._

FT‐IR spectrum of α‐Fe_2_O_3_/CuO/CuFe_2_O_4_ nanocomposite is shown in Figure 4, where two sharp peaks at 552 cm^−1^ and 371 cm^−1^ and a small peak at 2346 cm^−1^. By analyzing the previously published works, the peak on 552 cm^−1^ is for Fe−O stretching vibration and 371 cm^−1^ is for O−Fe‐O bending vibration of α‐Fe_2_O_3_,^[82]^ peak 552 cm^−1^ is for M_tetra_−O bond and 371 cm^−1^ is for M_octa_−O bond of CuFe_2_O_4_[^71^, ^77^] and peak 552 cm^−1^ for the Cu−O bond of CuO.^[69]^ As it synthesized as a nanocomposite particle, they combined to give these two peaks. The small absorption band at around 2346 cm^−1^ is due to the O=C=O stretching vibration of CuO^[83]^ It has no other external peak, which proves there are no impurities present.

### 3.4 UV‐Visible Spectroscopy

**Figure 5:**
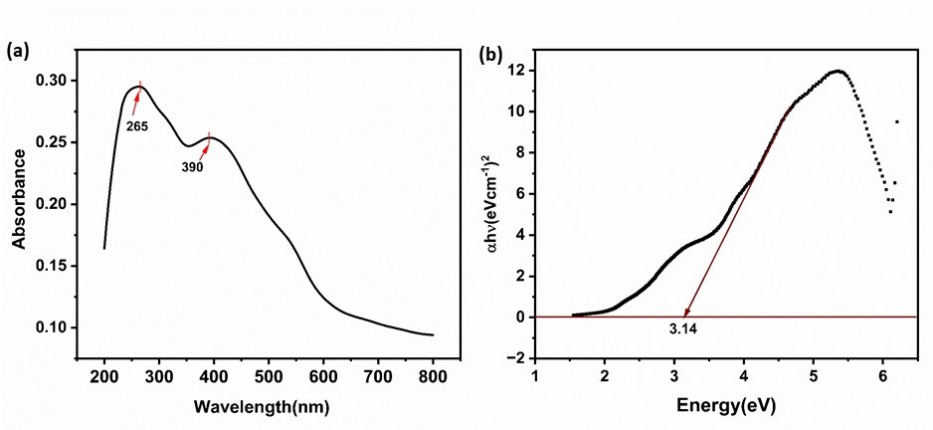
(a) UV‐visible spectrum of α‐Fe_2_O_3_/CuO/CuFe_2_O_4_ nanocomposite, (b) Tauc plot for band gap of α‐Fe_2_O_3_/CuO/CuFe_2_O_4_ nanocomposite.

The optical properties of the α‐Fe_2_O_3_/CuO/CuFe_2_O_4_ nanocomposite were studied using UV‐visible spectroscopy, with the absorption spectrum of Figure 5a. The value of the energy band gap is interrelated to the electron excited from the valence band to the conduction band, which is highly significant for the optical study of the material. The optical band gap is calculated using Tauc's relation, which is given in Equation 6.
(6)

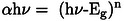




Where n is the pure numbers refer to the various kinds of electronic transitions, h is the Planck constant, E_g_ is the energy gap and ν is the photon frequency. The transitions are directly allowed, indirectly allowed, directly forbidden, and indirectly forbidden for n =1/2 , 2,3/2 and 3, respectively. Extrapolating linear segment of the curves to give the value of the band gap as the intercept to the horizontal axis, where αE=0..^[84]^

As previously reported, the band gap of directly allowed transition for α‐Fe_2_O_3_ is 2.0 eV,^[85]^ CuO is 2.18 eV^[86]^ and tetragonal CuFe_2_O_4_ is 3.14 eV.^[87]^ In this current study, α‐Fe_2_O_3_/CuO/CuFe_2_O_4_ nanocomposites directly allowed electronic transition (n =1/2 ) was calculated, and its energy gaps were 3.14 eV as in Figure 5b.

### 3.5 Field Emission Scanning Electron Microscopic (FE‐SEM) & Energy Dispersive X‐Ray (EDX) Spectroscopic Analysis

**Figure 6:**
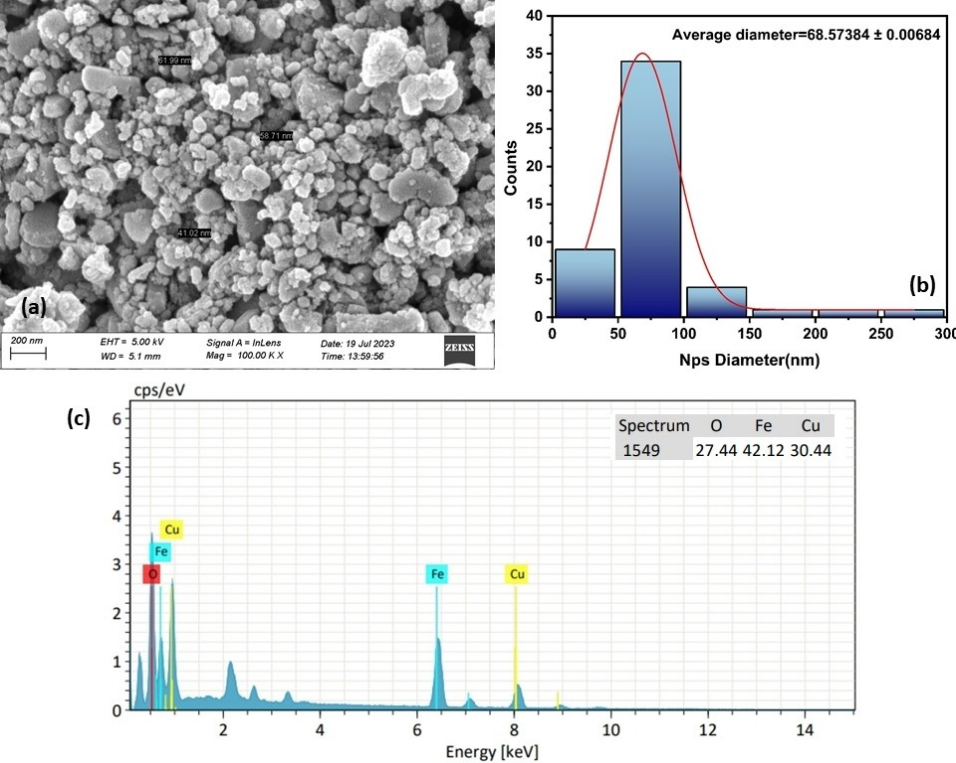
(a) SEM image of α‐Fe_2_O_3_/CuO/CuFe_2_O_4_ nanocomposite (b) Histogram of nanocomposite particles (c) EDX spectrum of nanocomposite.

Surface morphology analysis of α‐Fe_2_O_3_/CuO/CuFe_2_O_4_ nanocomposite was done using Field Emission Scanning Electron Microscopy (FE‐SEM), where the well‐distributed particles were observed (Figure 6). The average particle size was calculated to be 68.57 nm. Random 50 particles were calculated using ImageJ software size by distributions histogram to the log‐normal distribution function as reported.^[88]^

(7) $$f\;\left(D\right)=\left(\frac{1}{\sqrt{2\pi }.\;\sigma_{D}}\right)exp\left[\frac{{ln}^{2}\left(\frac{D}{D_{o}}\right)}{{2\sigma }^{2}}\right]$$

The elemental composition of these nanocomposites was investigated using EDX analysis. The estimated EDX spectrum confirms 42.12 % of iron (Fe) elemental species, 30.44 % of copper (Cu), and 29.75 % of oxygen (O) species.

### 3.6 Vibrating Sample Magnetometer (VSM)

**Figure 7:**
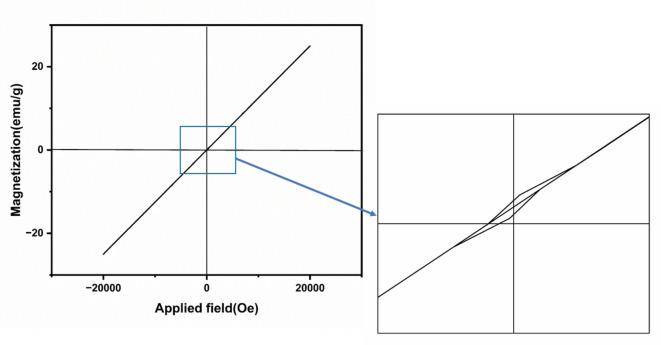
VSM graph of α‐Fe_2_O_3_/CuO/CuFe_2_O_4_ nanocomposite with an enlarged portion of the magnetic field line.

**Figure 8:**
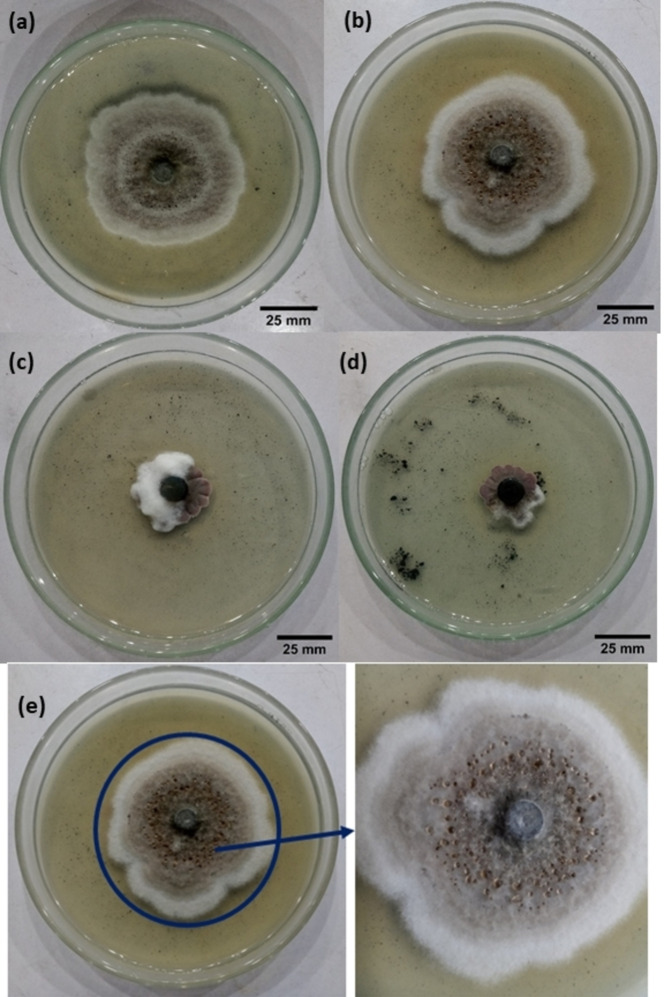
Antifungal effect of α‐Fe_2_O_3_/CuO/CuFe_2_O_4_ (a) sample‐1 against Alternaria spp. (b) sample‐2 against Alternaria spp. (c) sample‐1 against Bipolaris sorokiniana (d) sample‐2 against Bipolaris sorokiniana (e) Enlarged portion of Alternaria spp. toxin release against sample 2.

Figure 7 shows the magnetic properties of the synthesized α‐Fe_2_O_3_/CuO/CuFe_2_O_4_ nanocomposite. A straight magnetic field line was observed here in the plot. Similar straight lines were observed before for each individual α‐Fe_2_O_3_,^[37]^ CuO^[36]^ and CuFe_2_O_4_.^[89]^ The applied magnetic field (H) progressively rises from 20 K Oe to −20 K Oe at room temperature. By plotting the magnetization (emu/g) against the applied magnetic field, H (Oe) yielded the magnetic hysteresis loop, from which the saturation magnetization (M_S_), magnetic remanence (M_R_), and coercivity (H_C_) were studied. Figure 8b shows magnetic loop was visible when a portion of the magnetic field line enlarged. In Figure 8b, the hysteresis curve shows a narrow loop that represents a soft magnet.^[90]^ A comparison between the prepared composite and the previously repoted composite is shown in Table 3.

**Table 3:** Comparison of magnetic parameters of α‐Fe_2_O_3_/CuO/CuFe_2_O_4_ nanocomposite with nanoparticles reported earlier.

Compounds	MS (emu/g)	MR (emu/g)	HC (Oe)	References
α‐Fe_2_O_3_	3.2	1.2	Approach to 0	^[91]^
Fe_3_O_4_	28.5	0.07	1.5	^[92]^
	42	0.18	2.5	
	54.2	0.98	5	
CuO	0.0554 (±0.05)	0.00066 (±0.05)	5.7182 (±0.05)	^[93]^
CuFe_2_O_4_	37.89	0.85	15	^[94]^
α‐Fe_2_O_3_/CuO/CuFe_2_O_4_	25.14	0.86	6.79	This work

### 3.7 Antifungal Effect Of Α‐Fe_2_O_3_/Cuo/Cufe_2_o_4_ Nanocomposite

**Figure 9:**
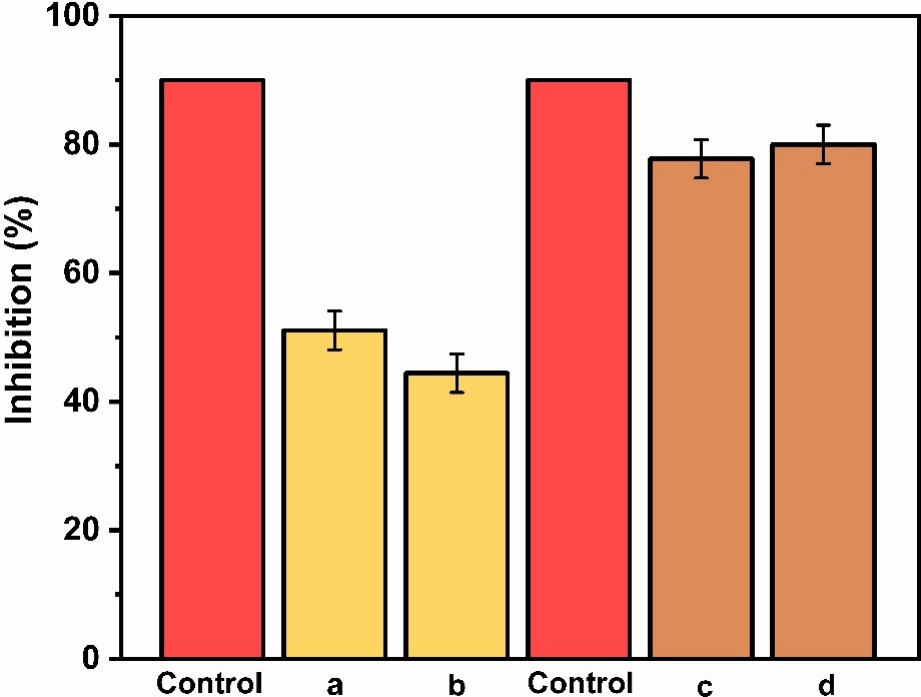
In vitro mycelial growth inhibition against fungi's.

The inhibitory activity of α‐Fe_2_O_3_/CuO/CuFe_2_O_4_ nanocomposite was investigated against *Alternaria spp*. and *Bipolaris sorakiniana* after 9 days, and the outcomes are shown in Figure 8 (a‐d). It was also noticed that the nanocomposite created a biologically stress environment for Alternaria spp. leading to the production of mycotoxins as a protective response (Figure 8e). It was seen that propiconazole fungicide control had 90 % inhibition against both fungicides, while the activity against Alternaria spp. was 51.11 % for sample 1 and 44.44 % for sample 2. The activity against Bipolaris sorakiniana for sample 1 is 77.77 %, and for sample 2 is 80 % **(**Figure 9). So this nanocomposite most effective against *Bipolaris sorakinana*.

*Alternaria spp*. are well‐known sources of more than 70 toxins of which a few are reported to have major phytotoxic,^[95]^ cytotoxic, mutagenic, carcinogenic, and genotoxic effects.^[96]^ Conversely, alternariol, one of the mycotoxins, has potential as a pharmacological and chemotherapeutic agent.^[97]^

### 3.8 Antioxidant Activity

**Figure 10:**
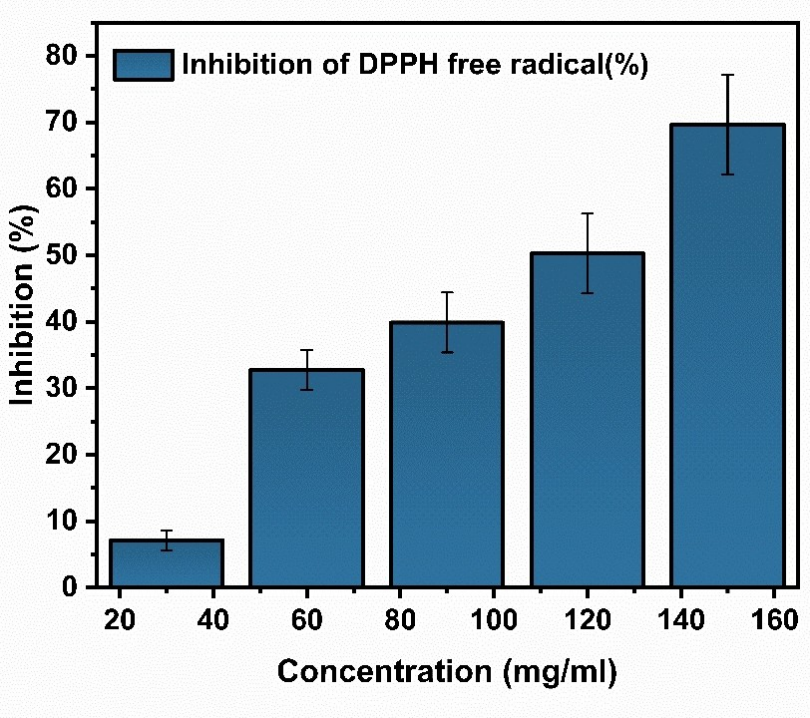
Dose‐dependent inhibition of DPPH activity of α‐Fe_2_O_3_/CuO/CuFe_2_O_4_ nanocomposite increased with concentrations.

Figure 10 reveals the antioxidant activity of α‐Fe_2_O_3_/CuO/CuFe_2_O_4_ nanocomposite, which gradually increases from 30 mg/ml to 150 mg/ml concentration. This sample exhibited 50 % inhibition (IC_50_) at a concentration of 109.88 μg/ml. Maximum DPPH free radical scavenging antioxidant activity was found at a 150 μg/ml concentration, reaching an antioxidant capacity of 69.61 %.

Many studies have proven antioxidant nanoparticles efficiently prevent cell damage from reactive oxygen species (ROS), can improve cell viability after ischemia‐reperfusion injury, and have promising antioxidant therapeutic effects in several oxidative stress‐related diseases, including neuronal injuries, wound healing, dry eye disease, and ulcerative colitis.[^98^, ^99^, ^100^] So α‐Fe_2_O_3_/CuO/CuFe_2_O_4_ nanocomposite has significant antioxidant properties making it a promising antioxidant in this approach.

### 3.9 pH_pzc_ Analysis

**Figure 11:**
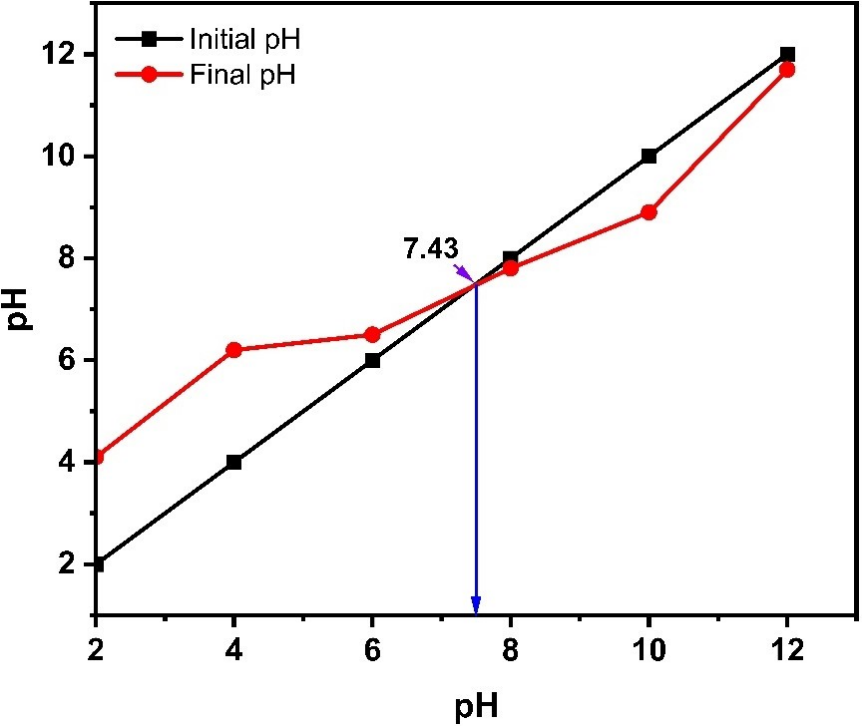
pH_PZC_ (point of zero charge) analysis

The isoelectric point, also referred to as PZC (point of zero charge), is the pH at which the particles in suspension have a net charge of zero and no mobility in the electric field. At pH_PZC_, the charge developed is neutral. From the obtained results, it is noteworthy that the surface charge of α‐Fe_2_O_3_/CuO/CuFe_2_O_4_ nanocomposite is positively charged at a pH lesser than the obtained PZC value of 7.43 (Figure 11).

### 3.10 Drug Loading

**Figure 12:**
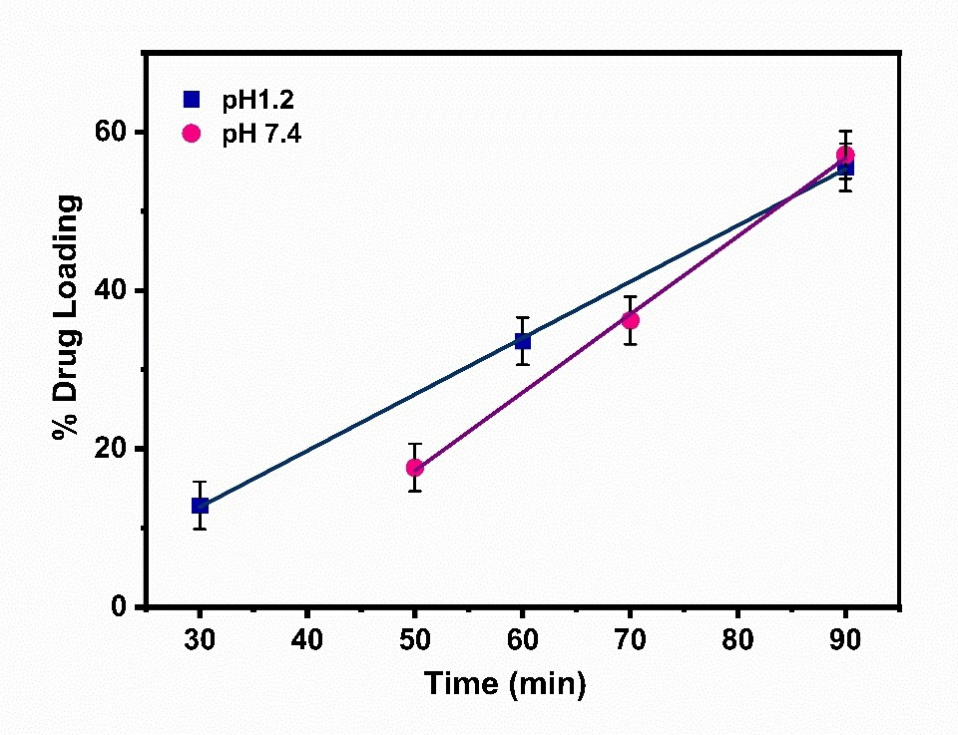
Drug loading profile of % of drug loading vs time

After 90 minutes, the percentage of loading of α‐Fe_2_O_3_/CuO/CuFe_2_O_4_ nanocomposite in pH 1.2 reached 55.53, while at pH 7.4 it reached 57.83 (Figure 12).

These results show the high drug loading efficiency of this nanocomposite which helps from frequent administration, costs, and increased side effects. This pH‐dependent drug loading offers precise control over doses, maintaining solubility and stability, and tuning stimuli sensitivity in combination therapies, and drug‐eluting medical implants.[^101^, ^102^]

### 3.11 Drug Release

**Figure 13:**
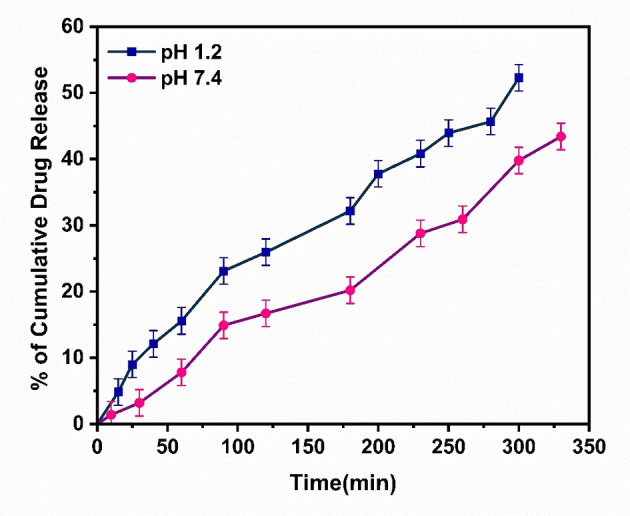
Drug release profile of % of cumulative drug release vs time

The percentage of cumulative drug release of α‐Fe_2_O_3_/CuO/CuFe_2_O_4_ nanocomposite at pH 7.4 after 300 min is 52.3, and the percentage of cumulative Drug Release at pH 1.2 after 330 min is 43.41. So a fast release is observed at pH 7.4 (Figure 13).

According to Carrales‐Alvarado et al.2014, in the pH range of 4 to 12, neutral pure metronidazole drug predominates, followed by cationic species for pH less than 4 and anionic species for pH greater than 12.^[103]^ The surface charge of α‐Fe_2_O_3_/CuO/CuFe_2_O_4_ nanocomposite is also positive <7.43 (calculated from pH‐PZC analysis). At pH 7.4, both are neutral. So, the surface interaction between the drug and nanocomposite is affected by the pH 1.2 because both are cationic. The interaction is comparatively weak (Table 4) here, so it loads less and gives a faster release at pH 1.2 than at pH 7.4.

**Table 4:** Statistical data of the regression equations for the drug loading in α‐Fe_2_O_3_/CuO/CuFe_2_O_4_ nanocomposite in different pH.

Parameter	pH 1.2	pH 7.4
Final % of loading	55.53	57.83
Coefficient of determination (R^2^)	0.99974	0.99883

### 3.12 Drug Release Kinetics

**Figure 14:**
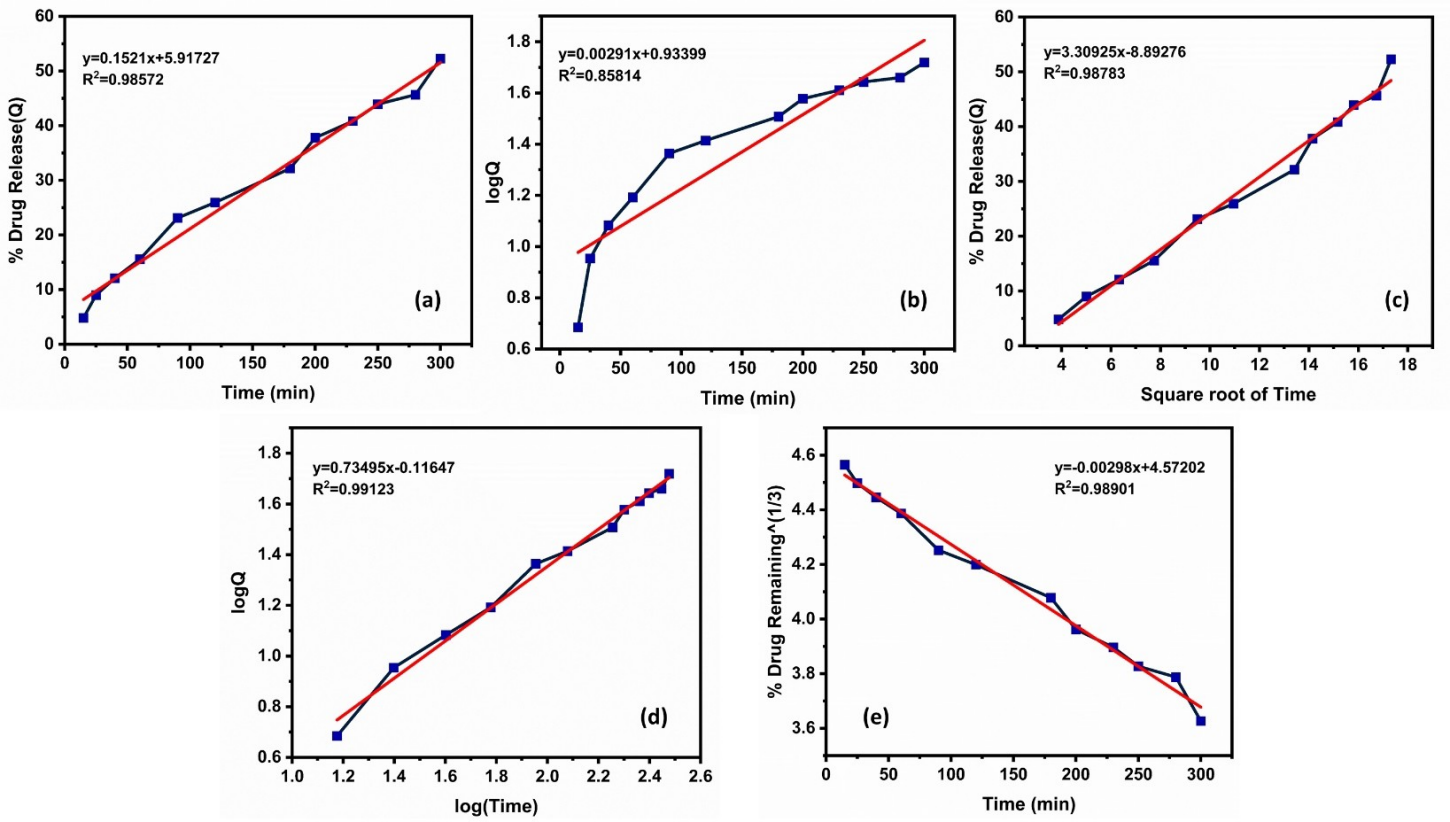
At pH 1.2 (a) Zero order kinetics (b) First order kinetics (c) Higuchi release profile (d) Korsmeyer–peppas kinetic model (e) Hixson‐crowell model

**Figure 15:**
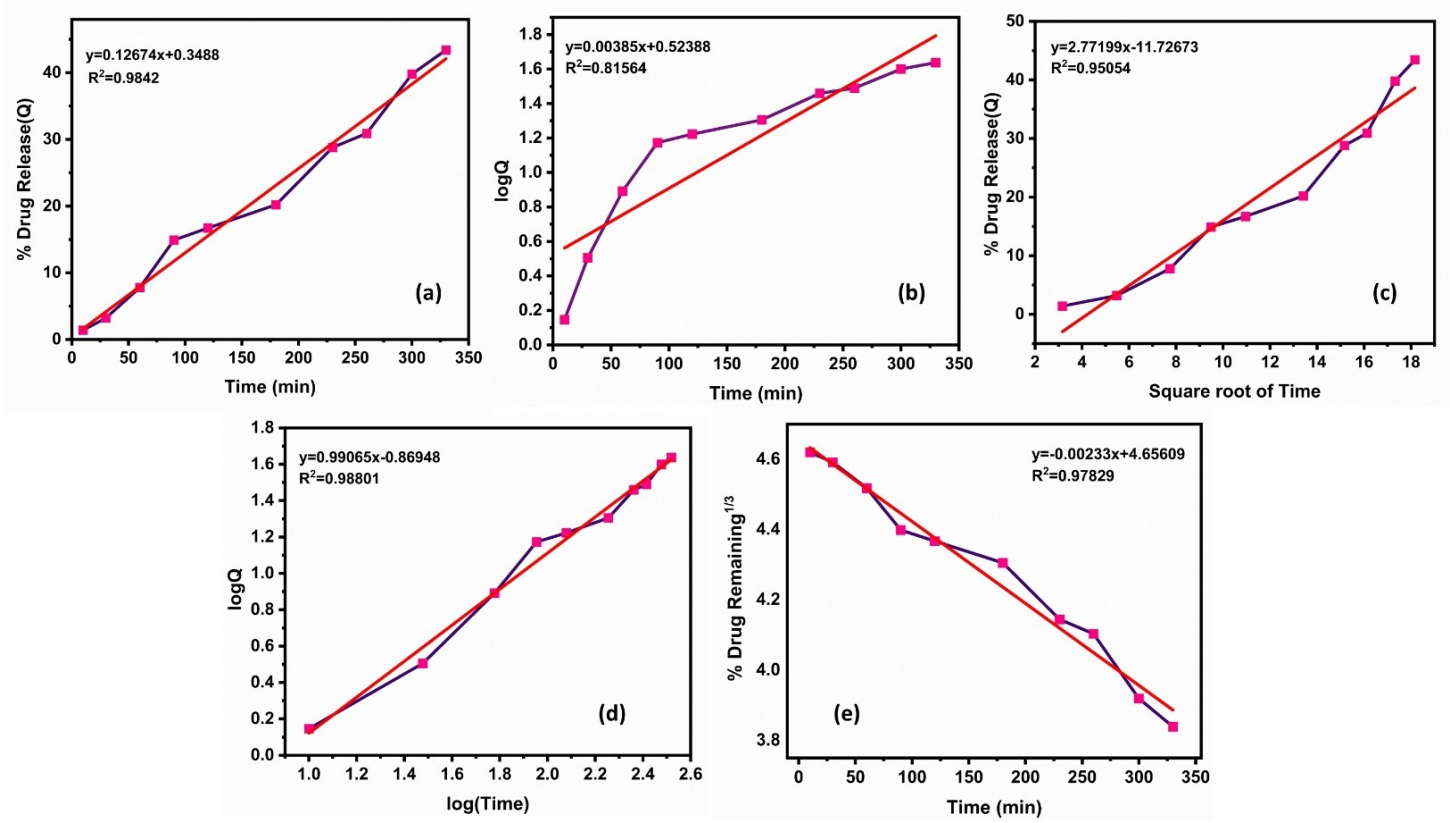
At pH 7.4 (a) Zero order kinetics (b) First order kinetics (c) Higuchi release profile (d) Korsmeyer–peppas kinetic model (e) Hixson‐crowell model.

Here, the Korsmeyer‐Peppas model is the best‐fitted kinetics (Table 5) for both pHs. For pH 1.2 (Figure 14), the n value is 0.73495, so it is anomalous (non‐Fickian) diffusion, and for pH 7.4 (Figure 15), super case‐II transport shows because the n value is 0.99065.

**Table 5:** The release kinetics evaluation at pH 1.2 and pH 7.4.

	Constants		Coefficient of determination (R2)	
Kinetic Models	**pH 1.2**	**pH 7.4**	**pH 1.2**	**pH 7.4**
Zero order kinetic model	0.1521 mg ml^−1^ min^−1^	0.12647 Mg ml^−1^ min^−1^	0.98572	0.9842
First order kinetic model	0.0067 min^−−1^	0.00886 min^−1^	0.85814	0.81564
Higuchi release model	3.30925 (mg ml^−1^ min^−1/2^)	2.77199 (mg ml^−1^ min^−1/2^)	0.98783	0.95054
Korsmeyer–Peppas model	‐0.11647 (mg ml^−1^ min^−1/n^) n=0.73495	‐0.86948 (mg ml^−1^ min^−1/n^) n=0.99065	0.99123	0.98801
Hixson–Crowell model	‐0.00298 (mgml^−1^)^1/3^ min^−1^	‐0.00233 (mgml^−1^)^1/3^ min^−1^	0.98901	0.97829

## 4 Conclusions

In this study, a sol‐gel auto‐combustion technique was employed to synthesize α‐Fe_2_O_3_/CuO/CuFe_2_O_4_ nanocomposite using a greener precursor, *Centella Asiatica*. The synthesized nanocomposite was characterized by XRD, FTIR, UV‐Vis, SEM, EDX, and VSM analyses. The nanocomposite exhibited excellent antifungal activity and antioxidant properties, demonstrating its physiological safety as a nanocarrier. Physiochemical properties such as encapsulation efficiency and in vitro release profiles varied with changes in pH variation. Based on these findings, the synthesized nanocomposite shows potential as a drug delivery system (DDS).

There are several promising directions for future research on Fe_2_O_3_/CuO/CuFe_2_O_4_ nanocomposite. Despite being a common stimulus in smart DDS, pH still requires a combination with other stimuli, such as temperature and redox, to obtain exceedingly exact and targeted release at the intended target sites. Metronidazole was used as a model drug for testing, although different drugs may yield varying results. Further studies should be carried out on Alternaria spp. mycotoxins to establish maximum residue limit standards and understand their toxicity mechanisms. To guarantee that this nanocomposite is practically employed, future research should concentrate on the investigation of clinical translation.

## Author Contributions

**Fahmida Akter Sahara**: Validation, Investigation, Methodology, Formal analysis, Software, Writing‐original Draft, **Dr. Mst. Sabiha Sultana**: Antifungal application guideline, **Md. Khairul Amin**: Writing‐Review & Editing, **Dr. Muhammad Shamim Al Mamun**: Writing‐Review & Editing, **Palash Kumar Dhar**: Fund acquisition, Project administration, Writing‐Review & Editing, **Sagar Kumar Dutta**: Conceptualization, Visualization, Methodology, Software, Writing‐Review & Editing, Supervision.

## Conflict of Interests

No conflict of interest has been reported by the authors.

5

## Data Availability

The data that support the findings of this study are available from the corresponding author upon reasonable request.
